# Development of an LC-MS/MS method to quantitatively analyze escitalopram and its metabolites with application in liver and placenta microsome metabolism

**DOI:** 10.3389/fphar.2025.1714686

**Published:** 2025-12-12

**Authors:** Yifei Shen, Bingyi Yao, Yuanqing Guo, Yujia Yang, Chenmeizi Liang, Junze Huang, Yuanjin Zhang, Xin Wang

**Affiliations:** Changning Maternity and Infant Health Hospital and School of Life Sciences, Shanghai Key Laboratory of Regulatory Biology, East China Normal University, Shanghai, China

**Keywords:** escitalopram, human placenta microsomes, LC-MS/MS, Liver microsomes, metabolism

## Abstract

**Background:**

Escitalopram (SCT), a highly effective and low-risk antidepressant, is widely used in the clinical treatment of depression. It undergoes two N-demethylation steps, producing its primary metabolites, S-demethylcitalopram (S-DCT) and S-didemethylcitalopram (S-DDCT).

**Purpose:**

This study aimed to develop and validate an LC-MS/MS method to simultaneously detect SCT, S-DCT, and S-DDCT, and then apply it to study metabolism in different microsomes, comparing SCT metabolism in rat liver microsomes (RLM), human liver microsomes (HLM), and human placenta microsomes (HPM).

**Methods:**

This study developed a reliable LC-MS/MS method for the simultaneous detection of escitalopram and its metabolites in various microsomal systems. The method was fully validated in rat liver microsomes and then was applied into human liver and placenta microsomes. The method was further applied to investigate escitalopram metabolism in different microsomal systems, notably for the first time in human placenta microsomes.

**Results:**

LC-MS/MS method provides high specificity, good stability, and excellent extraction recovery. Both intra-day and inter-day accuracy (% RE) and precision (% RSD) were within ±15%, with no significant matrix effects observed. The metabolic rates of escitalopram and its metabolite levels differed among rat liver, human liver, and human placenta microsomes, likely due to variations in the types and activities of drug-metabolizing enzymes present in these systems.

**Conclusion:**

A sensitive and reliable LC-MS/MS method was developed and validated for the quantitative analysis of SCT, S-DCT, and S-DDCT in RLM. Subsequently, the method was applied to the study of HLM and HPM. Incubation experiments using these microsomal systems showed that all three types of microsomes, including HPM, could metabolize SCT.

## 1 Introduction

**FIGURE 1:**
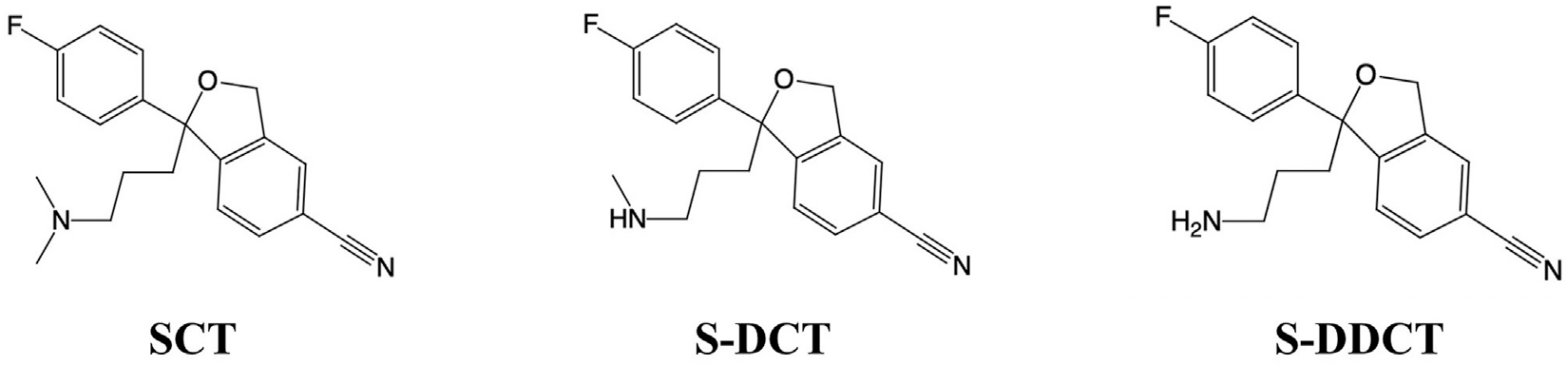
Chemical structure of escitalopram (SCT), S-demethylcitalopram (S-DCT), and S-didemethylcitalopram (S-DDCT).

Major depressive disorder (MDD) is a mood disorder marked by a significant low mood or loss of energy lasting at least 2 weeks and significantly impacting daily life (Battle, 2013; Becker et al., 2016). Perinatal depression is one of the most common psychological disorders during pregnancy. About 20% of pregnant women experience some depressive symptoms before giving birth, 15% suffer from major depressive disorder, and 17% develop postpartum depression (Pearlstein, 2015). Approximately 7.5% of women are exposed to at least one type of antidepressant (Mitchell et al., 2011). Currently, selective serotonin reuptake inhibitors (SSRIs) are widely used to treat depression because of their effectiveness and low risk, making them the preferred therapy (Cipriani et al., 2018). They increase extracellular serotonin levels by inhibiting the reuptake of serotonin by presynaptic cells, allowing serotonin to bind to postsynaptic receptors (Pastoor and Gobburu, 2014). Among these, escitalopram ((S)-(+)-1-[3-(dimethylamino) propyl]-1-(p-fluorophenyl)-5-phthalancarbonitrile oxalate, Figure 1) has become a common choice in clinical practice due to its high selectivity, relatively low potential for drug interactions, and good tolerability (Cipriani et al., 2018; Cipriani et al., 2009; Sanchez et al., 2014). Moreover, patients receiving treatment with escitalopram had different protein and enriched pathways, and these changes were associated with MDD (Huan et al., 2021). However, other studies demonstrated a possible increase of some inflammatory cytokines after taking escitalopram (Zhou et al., 2021). In summary, compared to other SSRIs, there is relatively little research on escitalopram. As a common clinical choice, clarifying its metabolism is crucial (Rethorst et al., 2017).

Escitalopram (SCT) is metabolized to S-demethylcitalopram (S-DCT, Figure 1) and further to S-didemethylcitalopram (S-DDCT, Figure 1) through two N-demethylation steps (Rao, 2007; Aronson and Delgado, 2004). CYP3A4, CYP2C19, and CYP2D6 facilitate the first demethylation, while the second is mainly mediated by CYP2D6 (Rao, 2007; von Moltke et al., 2001). S-DCT, the primary metabolite, makes up about one-third of SCT (Rao, 2007). Some studies suggest that SCT is a weak inhibitor of CYP2D6 (Noehr-Jensen et al., 2009), and poor CYP2C19 metabolizers have higher plasma concentrations of SCT and lower levels of metabolites (Herrlin et al., 2003). Additionally, SCT and its metabolites can cross the placental barrier and enter the fetal bloodstream (Ewing et al., 2015). However, it remains uncertain whether the placenta can metabolize SCT.

Currently, LC-ESI-MS/MS and GS-MS/MS techniques are used to detect SCT in human urine, plasma, and other biological samples (Badulla et al., 2020; Guan et al., 2020; Canbolat et al., 2019). There are also some LC-MS/MS methods for separating and measuring SCT and its metabolites (de Sousa et al., 2022; Johansen, 2017). However, no method exists for quantifying SCT and its main metabolites in the microsomal metabolism system. The microsomal metabolism system, as the core model for *in vitro* drug metabolism research, is due to its ability to simulate key processes of drug metabolism *in vivo* to the greatest extent possible, while also possessing advantages such as experimental controllability, stability, and economy. This study aimed to develop and validate an LC-MS/MS method to simultaneously detect SCT, S-DCT, and S-DDCT, and then apply it to study metabolism in different microsomes, comparing SCT metabolism in rat liver microsomes (RLM), human liver microsomes (HLM), and human placenta microsomes (HPM).

## 2 Materials and methods

### 2.1 Materials

Escitalopram (purity 98%) was purchased from Macklin Biochemical Technology Co., Ltd. (Shanghai, China). S-DCT (purity 99%) and S-DDCT (purity 99%) were purchased from ChemStrong Scientific Co., Ltd. (Shenzhen, China). Dexamethasone (purity 98%) was bought from Alfa Aesar (Ward Hill, MA, United States). Acetonitrile and methanol (HPLC grade) were from Fisher Chemicals (Leicester, United Kingdom), and formic acid and acetic acid (HPLC grade) were bought from TEDIA (Ohio, United States). All the distilled water was purified through a Millipore system (Milli-Q, Massachusetts, United States). Pooled HLM (n = 150) were obtained from BioIVT (New York, United States) and stored at −80 °C for future use.

### 2.2 Preparation of RLM and HPM

Pooled RLM (n = 10) were prepared previously in our laboratory (Liang et al., 2025). The animal study was approved by Experimental Ethics Committee of East China Normal University. The study was conducted in accordance with the local legislation and institutional requirements. Human placentas were collected from twenty healthy donors after full-term pregnancies. All procedures involving human subjects were carried out under the principles of the Declaration of Helsinki and the Ethical Review Methods for Biomedical Research Involving Humans, adopted by the Family Planning Commission of the People’s Republic of China. All experiments and protocols received approval from the Medical Ethics Committee of Shanghai Changning Maternity and Infant Health Hospital. The participants provided their written informed consent to participate in this study. The microsome preparation method followed the procedures described in our previous studies (Fu et al., 2023), involving two rounds of centrifugation at different speeds. After collecting the microsomes, the BCA assay was used to determine protein concentration. Finally, the samples were stored at −80 °C for future use.

### 2.3 LC-MS/MS condition

The experiment was performed using an Agilent 1290 liquid chromatography system, which included a high-speed pump (G7120A), an autosampler (G7167B), and a column oven (G7116B). SCT, S-DCT, S-DDCT, and dexamethasone (internal standard, IS) were detected by an Agilent 6470 triple-quadrupole mass spectrometer (Agilent Technologies, United States) equipped with an Agilent Jet Stream electrospray ionization (ESI) source. They were separated using a Phenomenex Kinetex XB-C18 column (100 × 3.00 mm, 2.6 μm).

The optimized MS conditions are as follows for positive-ionization mode: gas temperature, 200 °C; collision gas (N2), 1.6 MPa; gas flow, 6 L/min; sheath gas temperature, 350 °C; nebulizer pressure, 25 psi; sheath gas flow, 12 L/min. The mobile phase comprises A (water with 0.1% formic acid and 1 mM ammonium formate) and B (acetonitrile with 0.1% formic acid). The separation of all the substances were conducted at a flow rate of 0.3 mL/min and the column temperature was kept at 30 °C.

### 2.4 Preparation of calibration samples and quality control samples

SCT, S-DCT, and S-DDCT were first dissolved in dimethyl sulfoxide to 3 mg/mL and stored at 4 °C. For preparing the linearity working solutions, SCT was diluted to 300, 500, 1000, 3000, 5000, and 10,000 ng/mL, while S-DCT and S-DDCT were diluted to 10, 30, 100, 300, 500, and 1,000 ng/mL with acetonitrile containing 0.1% formic acid. Similarly, the working solutions for quality control (QC) samples were 300, 400, 5000, and 8000 ng/mL for SCT and 10, 20, 500, and 800 ng/mL for S-DCT and S-DDCT. To prepare 10-fold diluted solutions, 10 μL of the working solution was added to 90 μL of the microsomal incubation system. Additionally, dexamethasone was weighed and dissolved to 250 ng/mL in acetonitrile containing 0.1% formic acid for future use.

### 2.5 Sample preparation

After preparing the calibration and QC samples, 200 μL of pre-cooled acetonitrile was added. The IS was added prior to the protein precipitation step to correct for variability throughout the sample preparation and analysis process. Each sample was then thoroughly vortexed and centrifuged at 12,000 g and 4 °C for 15 min. An aliquot of 70 μL of the supernatant was subsequently used for LC-MS/MS analysis.

### 2.6 Method validation

#### 2.6.1 Specificity

The specificity was assessed by comparing the chromatograms of the blank RLM matrix, RLM with three analytes at the lower limit of quantification (LLOQ), and the SCT incubation mixture in RLM. For the method to be considered specific, the retention times of analytes and IS should be consistent, with no interfering peaks present.

#### 2.6.2 Linearity and LLOQ

Calibration curves were generated by plotting the analyte-to-IS response ratio against the corresponding analyte concentrations. Linear regression was used to fit the calibration curves for all three analytes, with weighting factors of 1/x or 1/x^2^ as appropriate. It was required that the *R* ^2^ values be greater than 0.99 and the signal-to-noise ratio (S/N) exceed 10.

#### 2.6.3 Accuracy and precision

Intra-day accuracy and precision were evaluated by analyzing LLOQ, low, medium, and high QC samples within a single day. For inter-day accuracy and precision, the QC samples were analyzed over three consecutive days. Accuracy was expressed as the percentage relative error (% RE), while precision was expressed as the percentage relative standard deviation (% RSD). According to FDA guidelines, the accuracy and precision of LLOQ should be within ±20%, while the other QC samples should be within ±15% (Chen et al., 2021; Fu et al., 2023; Huang et al., 2021).

#### 2.6.4 Matrix effect and extraction recovery

The matrix effect was evaluated by comparing the peak areas of QC samples for three analytes and IS in microsomal incubation matrix with those in Tris/HCl buffer solution. Extraction recovery was measured by comparing the peak areas of QC samples with standard solutions at the same concentration. Specifically, acetonitrile should be added both before and after the analytes.

#### 2.6.5 Stability

The stability of QC samples was assessed under various conditions. In brief, all the samples were stored at room temperature for 4 h to evaluate short-term stability. The post-preparative storage stability was tested by keeping the processed samples in sampling vials for 24 h at room temperature. The samples were deemed stable if the % RE and % RSD fell within ±20% at the LLOQ level and within ±15% at low, medium, and high levels.

### 2.7 Microsomal incubation experiment

As described in our previous study (Fu et al., 2023), incubation experiments were conducted in various microsomal systems. In short, the mixture (100 μL) included different microsomes (1 mg/mL for RLM, 0.5 mg/mL for HLM, or 2 mg/mL for HPM), glucose 6-phosphate (G6P, 10 mM), glucose 6-phosphate dehydrogenase (G6PDH, 0.4 U/mL), and SCT (1–50 μM) in Tris/HCl buffer. The β-nicotinamide adenine dinucleotide phosphate (NADP, 1 mM) was added after a 5-min pre-incubation at 37 °C to start the reaction. To prevent denaturation and inhibition of the metabolizing enzymes, the organic solvents in the final incubation systems should not exceed 1% (v/v). All microsomal systems were incubated for 30 min, after which the samples were prepared following the procedure in Section 2.5.

### 2.8 Data analysis

All experiments were conducted using four independent biological replicates. All data were presented as mean ± SD. Kinetic parameters (K_m_ and V_max_) for SCT metabolism were determined by fitting the metabolite formation rate data to the Michaelis-Menten model using non-linear regression analysis in GraphPad Prism 9.0 (GraphPad Software Inc., United States). Comparisons across tissues were made using one-way ANOVA among three groups and *t*-test between two groups. Since the metabolism of SCT involved two steps of N-demethylation, to calculate its metabolic ratio, the concentration of S-DCT was calculated as: [(molar concentration of S-DCT + S-DDCT)/(molar concentration of SCT added) * 100%]. For the conversion of S-DCT to S-DDCT, it was calculated as: [(molar concentration of S-DDCT formed)/(molar concentration of S-DCT) * 100%]. The statistical power in each experiment was 0.95 and it was presented as *p < 0.05, **p < 0.01, and ***p < 0.001.

## 3 Results and discussion

### 3.1 Method development

A sensitive LC-MS/MS method was developed for the detection and separation of SCT, S-DCT, S-DDCT, and IS. Relevant detection parameters of the analytes are summarized in Table 1. For chromatographic separation, the mobile phase gradient started with 70% A and 30% B, then changed to 30%–40% B (0–3 min), 40% B (3–3.5 min), 40%–90% B (3.5–3.8 min), 90% B (3.8–5 min), 90%–30% B (5–5.2 min), and returned to 30% B (5.2–7 min).

**TABLE 1:** Mass spectrometry parameters of SCT, S-DCT, S-DDCT, and dexamethasone (IS).

Analytes	Polarity	Precursor ion (m/z)	Production (m/z)	Fragmentor (V)	Collision energy (eV)
SCT	ESI+	325.2	109.1	150	29
S-DCT	ESI+	311.1	109.1	120	36
S-DDCT	ESI+	297.1	109.1	90	31
Dexamethasone (IS)	ESI+	393.3	373.3	130	5

### 3.2 Method validation

#### 3.2.1 Specificity

**FIGURE 2:**
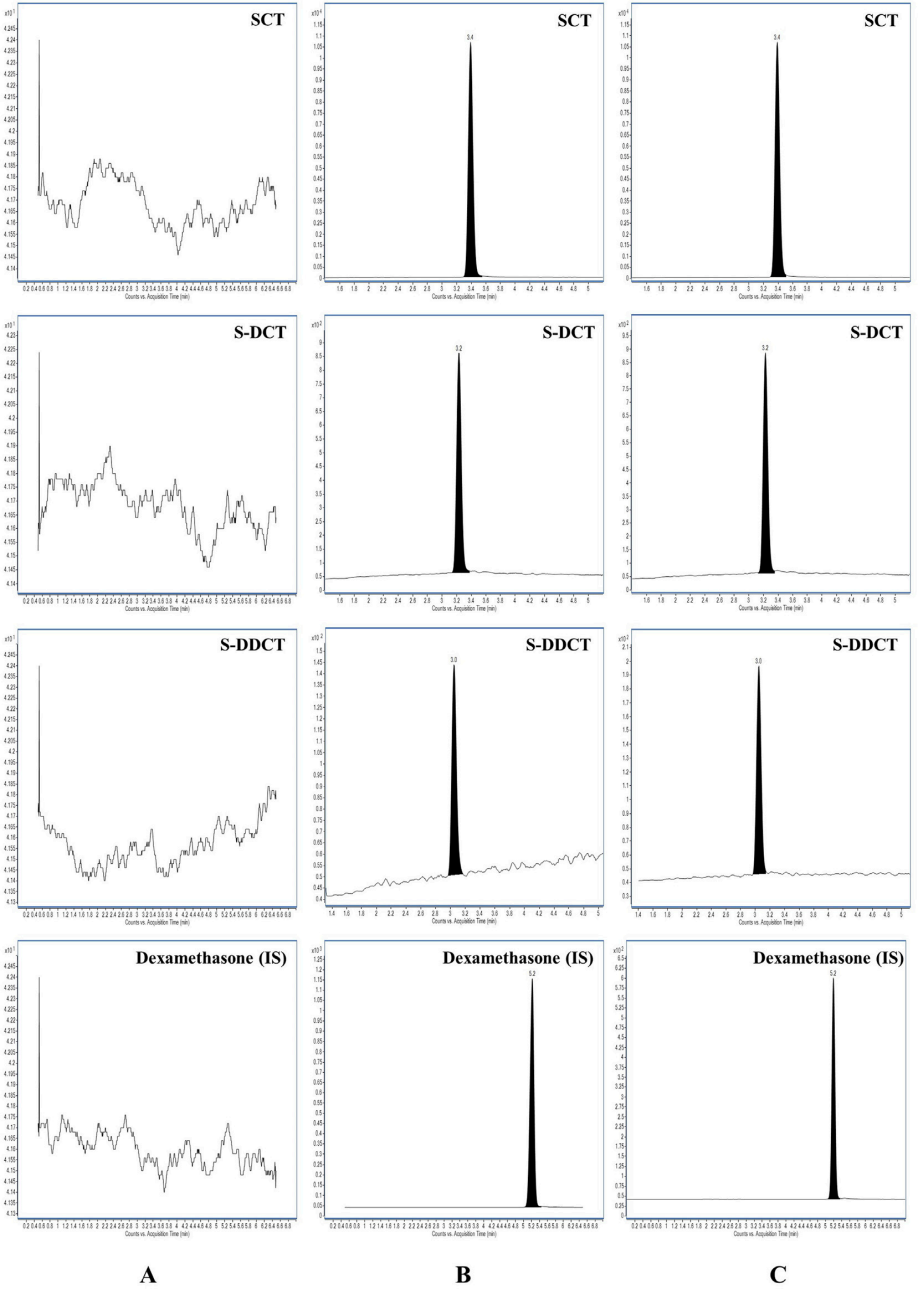
MRM chromatograms of SCT, S-DCT, S-DDCT, and dexamethasone (IS) in RLM, respectively. **(A)** Blank RLM matrix. **(B)** RLM matrix with SCT, S-DCT, S-DDCT, and IS at the LLOQ level. **(C)** SCT incubated in RLM. SCT, escitalopram; S-DCT, S-demethylcitalopram; S-DDCT, S-didemethylcitalopram; RLM, rat liver microsomes; LLOQ, lower limit of quantification.

As shown in Figure 2, SCT, S-DCT, S-DDCT, and IS were successfully detected and baseline-separated. The retention times for SCT, S-DCT, S-DDCT, and IS were 3.4, 3.2, 3.0, and 5.2 min, respectively. There were no interfering peaks in the blank matrix, and both the peaks at the LLOQ level and after incubation were well-defined and distinct.

#### 3.2.2 Linearity and LLOQ

The concentration of SCT ranged from 30 to 1,000 ng/mL (y = 25.205x + 1.968, weighing 1/x). S-DCT ranged from 1 to 100 ng/mL (y = 27.840x + 0.080, weighing 1/x), and S-DDCT ranged from 1 to 100 ng/mL (y = 5.727x + 0.027, weighing 1/x2). The LLOQ of SCT was 30 ng/mL, while S-DCT and S-DDCT had an LLOQ of 1 ng/mL. The *R* ^2^ values were all above 0.99, and the S/N ratios exceeded 10.

#### 3.2.3 Accuracy and precision

Samples at four levels (LLOQ, low, medium, and high) were analyzed to determine accuracy and precision. As shown in Table 2, the intra-day and inter-day accuracy (% RE) for all four levels were within ±15%, and the precision (% RSD) was less than 15% across all levels, which all conform to FDA guidelines.

**TABLE 2:** Intra-day and inter-day accuracy and precision of SCT, S-DCT, and S-DDCT in RLM.

Analytes	Nominal concentration (ng/mL)	Intra-day (n = 6)			Inter-day (n = 6)		
		Measured concentration (mean ± SD, ng/mL)	Accuracy (RE, %)	Precision (RSD, %)	Measured concentration (mean ± SD, ng/mL)	Accuracy (RE, %)	Precision (RSD, %)
SCT	30	26.80 ± 2.57	−10.67	9.57	25.66 ± 1.85	−14.48	7.22
	40	39.66 ± 5.25	−0.85	13.23	36.39 ± 4.65	−9.02	12.77
	500	550.03 ± 39.18	10.01	7.12	540.59 ± 29.40	8.12	5.44
	800	821.70 ± 40.67	2.71	4.95	787.01 ± 39.43	−1.62	5.01
S-DCT	1	1.13 ± 0.09	13.35	8.24	1.08 ± 0.08	7.61	7.04
	2	2.00 ± 0.10	−0.14	4.93	2.11 ± 0.15	5.46	7.14
	50	49.19 ± 4.67	−1.62	9.49	45.84 ± 3.74	−8.31	8.16
	80	69.63 ± 4.31	−12.96	6.18	73.14 ± 3.84	−8.56	5.24
S-DDCT	1	0.86 ± 0.11	−13.55	12.53	0.88 ± 0.07	−11.53	8.41
	2	1.85 ± 0.28	−7.33	14.91	1.80 ± 0.20	−9.89	11.14
	50	51.46 ± 2.57	2.93	5.01	50.73 ± 1.77	1.46	3.48
	80	78.08 ± 3.73	−2.40	4.78	76.15 ± 2.95	−4.79	3.88

#### 3.2.4 Matrix effect and extraction recovery

Matrix effects and extraction recoveries were assessed by comparing peak areas of samples prepared using different methods. In the RLM matrix (Table 3), the extraction recovery of three analytes ranged from 90.54% to 100.41%, with all % RSDs below 7%. Regarding matrix effects, the ratios ranged from 90.83% to 101.28%, indicating no significant matrix effect. Since the method was intended for testing in HPM and HLM, matrix effects in these matrices were also evaluated. As shown in Table 4, ratios in HPM ranged from 93.76% to 107.81% (RSD <6%), and in HLM ranged from 95.96% to 112.09% (RSD <10%), confirming no notable matrix effects in these systems.

**TABLE 3:** Extraction recovery and matrix effect of SCT, S-DCT, S-DDCT, and dexamethasone (IS) in RLM (n = 6).

Analytes	Nominal concentration (ng/mL)	Extraction recovery (%)	RSD (%)	Matrix effect (%)	RSD (%)
SCT	30	90.54	1.34	94.63	2.18
	40	93.27	2.09	97.25	1.7
	500	92.85	2.44	98.63	3.11
	800	97.03	3.6	97.06	4.49
S-DCT	1	96.57	2.32	99.12	1.81
	2	91.47	2.89	94.68	2.82
	50	95.37	1.77	99.61	4.12
	80	100.41	1.29	101.28	6.82
S-DDCT	1	91.87	3.96	92.15	3.04
	2	95	6.73	90.83	3.28
	50	95.31	1.44	99.03	4.01
	80	98.15	3.06	101.08	4.36
Dexamethasone (IS)	250	100.54	2.44	104.64	6.09

**TABLE 4:** Matrix effect of SCT, S-DCT, S-DDCT, and dexamethasone (IS) in HLM and HPM (n = 6).

Analytes	Nominal concentration (ng/mL)	HPM		HLM	
		Matrix effect (%)	RSD (%)	Matrix effect (%)	RSD (%)
SCT	30	107.81	1.8	99.88	2.44
	40	101.51	2.45	105.88	0.75
	500	103.9	5.18	112.09	5.99
	800	106.76	3.62	108.98	5.37
S-DCT	1	102.81	0.78	106.71	1.07
	2	102.75	2	110.97	1.21
	50	101.59	3.38	108.05	5.5
	80	101.88	7.92	100.02	6.73
S-DDCT	1	100.61	2.54	95.96	1.59
	2	93.76	1.74	99.06	1.97
	50	100.93	3.29	110.11	7.76
	80	102.01	5.1	109.02	9.22
Dexamethasone (IS)	250	96.08	9.6	91.68	9.79

#### 3.2.5 Stability

The analytes remained stable after 4 h (short-term stability) of storage at room temperature and after 24 h (post-preparative stability) in sampling vials, with relevant data summarized in Table 5. Since all the % RE values were within ±15% with no significant fluctuations, this indicates that the method and experimental conditions were suitable for all analytes and could meet basic standards.

**TABLE 5:** Stability of SCT, S-DCT, and S-DDCT in RLM (n = 6).

Stability mode	Analytes	Nominal concentration (ng/mL)	Measured concentration (mean ± SD, ng/mL)	Accuracy (RE, %)	Precision (RSD, %)
Short-term stability	SCT	30	27.41 ± 1.18	−8.64	4.29
		40	36.96 ± 0.89	−7.61	2.41
		500	519.23 ± 12.76	3.85	2.46
		800	707.51 ± 15.57	−11.56	2.21
	S-DCT	1	0.97 ± 0.05	−3.48	5.02
		2	2.05 ± 0.08	2.66	4.09
		50	51.70 ± 1.15	3.41	2.23
		80	69.44 ± 1.26	−13.21	1.82
	S-DDCT	1	1.00 ± 0.06	−0.31	5.68
		2	2.10 ± 0.08	5.18	3.82
		50	47.71 ± 1.08	−4.58	2.26
		80	70.10 ± 2.23	−12.38	3.18
Postpreparative stability	SCT	30	25.63 ± 1.02	−14.58	3.97
		40	35.66 ± 1.00	−10.86	2.82
		500	555.55 ± 14.62	11.11	2.63
		800	787.83 ± 21.23	−1.52	2.69
	S-DCT	1	1.06 ± 0.05	5.94	4.59
		2	2.29 ± 0.06	14.35	0.03
		50	44.49 ± 1.86	−9.27	4.18
		80	75.85 ± 1.69	−4.48	2.23
	S-DDCT	1	0.90 ± 0.03	−9.76	3.14
		2	1.78 ± 0.03	−11.21	1.61
		50	50.19 ± 1.25	0.38	2.51
		80	75.68 ± 2.40	−5.39	3.17

### 3.3 Application of the method to microsomal incubation experiments

**FIGURE 3:**
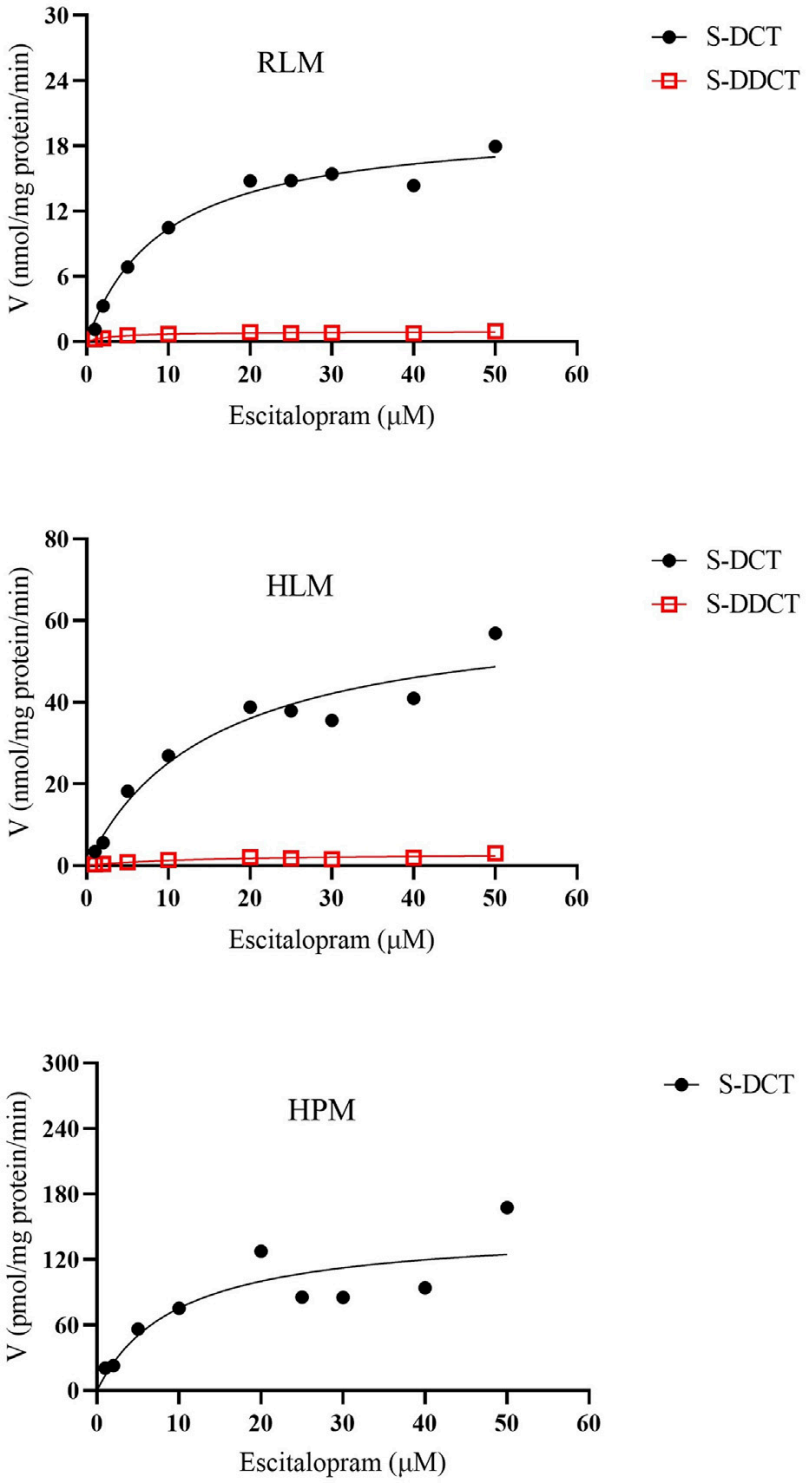
The Michaelis-Menten curves of escitalopram metabolism in RLM, HLM, and HPM. All data were displayed as the mean value of four experiments. S-DCT, S-demethylcitalopram; S-DDCT, S-didemethylcitalopram; RLM, rat liver microsomes; HLM, human liver microsomes; HPM, human placenta microsomes.

**FIGURE 4:**
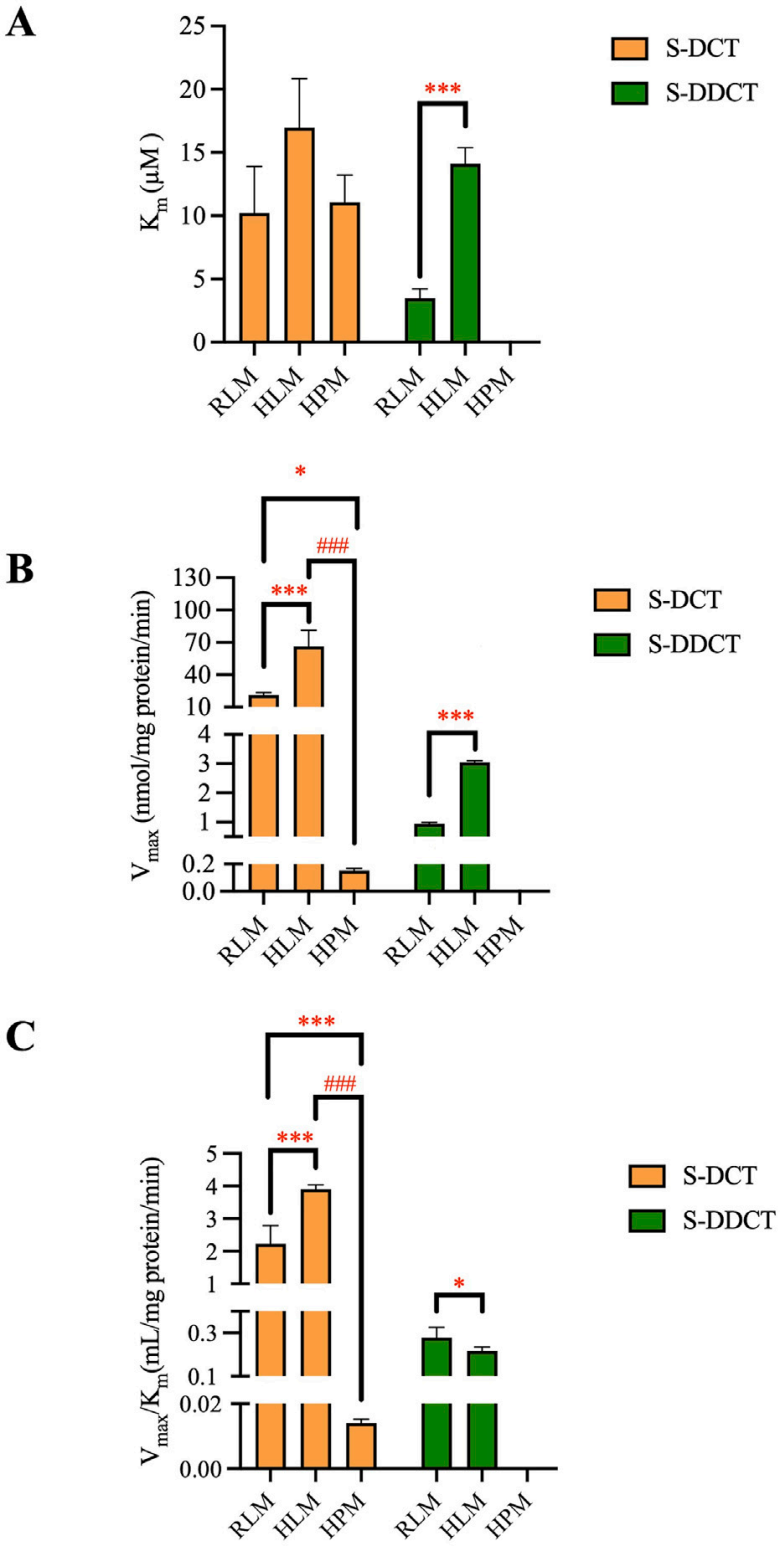
The kinetic analysis of escitalopram metabolism in RLM, HLM, and HPM. The K_m_ **(A)**, V_max_ **(B)**, and V_max_/K_m_ **(C)** values of S-DCT and S-DDCT in RLM, HLM, and HPM. All data were displayed as mean ± SD of four experiments. *p < 0.05, ***p < 0.001, and ^###^ *p* < 0.001. S-DCT, S-demethylcitalopram; S-DDCT, S-didemethylcitalopram; RLM, rat liver microsomes; HLM, human liver microsomes; HPM, human placenta microsomes.

**FIGURE 5:**
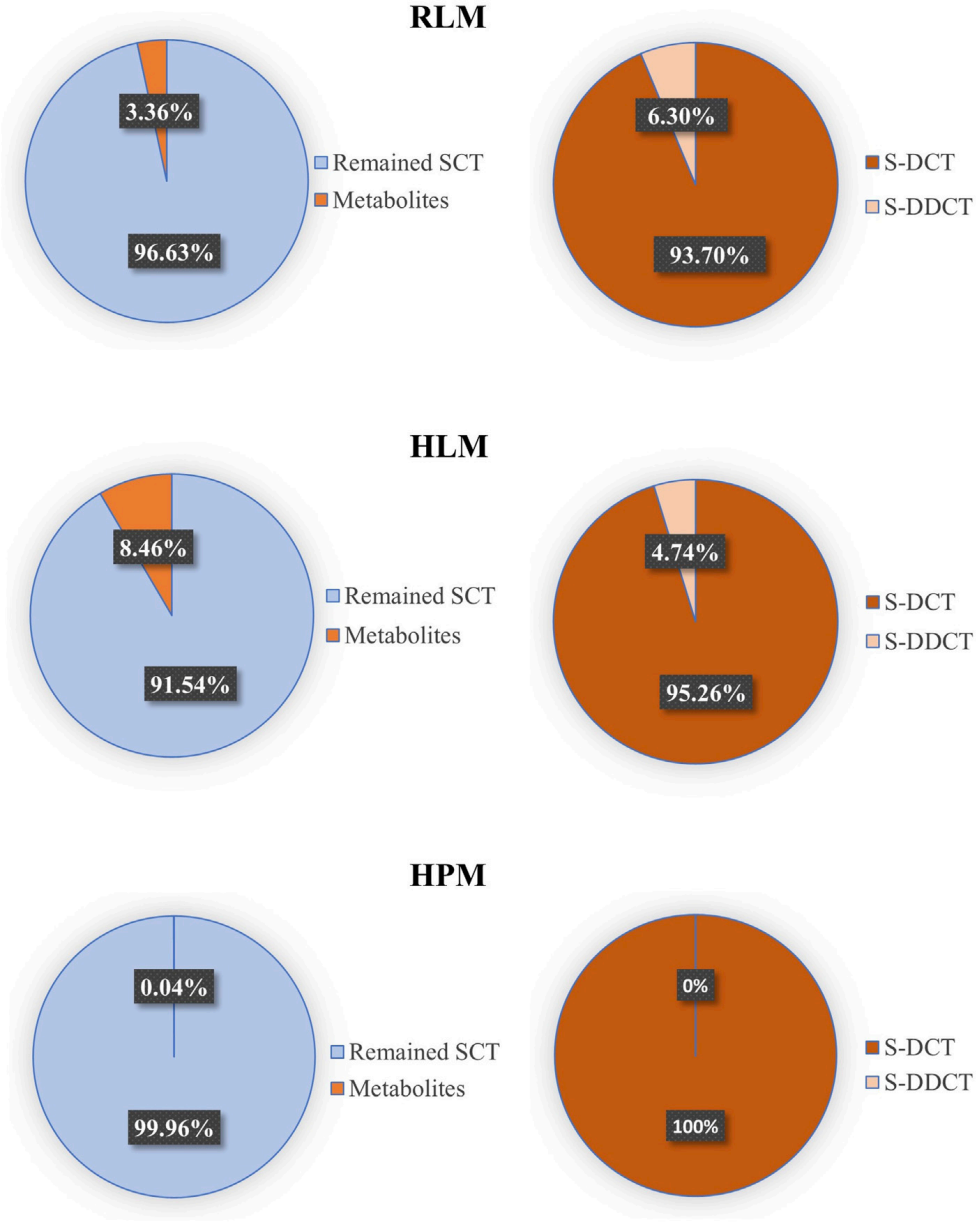
The proportion of major metabolites and the remained escitalopram in RLM, HLM, and HPM. SCT, escitalopram; S-DCT, S-demethylcitalopram; S-DDCT, S-didemethylcitalopram; RLM, rat liver microsomes; HLM, human liver microsomes; HPM, human placenta microsomes.

Based on the data presented above, the validated method accurately and precisely detected and separated SCT, S-DCT, and S-DDCT, with no significant matrix effect observed in RLM, HPM, or HLM. Therefore, it is suitable for use in incubation experiments across different microsomal systems. First, in RLM and HLM, both metabolites, S-DCT and S-DDCT, were detected, whereas only S-DCT was found in HPM, indicating species- and tissue-specific differences in SCT metabolism. The Michaelis-Menten curves for SCT metabolism are shown in Figure 3. Kinetic analysis revealed that in RLM, the V_max_ values for S-DCT and S-DDCT were 20.64 ± 1.19 and 0.95 ± 0.03 nmol/mg protein/min, respectively, while the corresponding K_m_ values were 10.23 ± 3.68 and 3.49 ± 0.74 µM (Figure 4). In HLM, the V_max_ values for the two analytes were 66.82 ± 6.23 and 3.05 ± 0.03 nmol/mg protein/min, with K_m_ values of 16.98 ± 3.87 and 14.12 ± 1.26 µM, respectively (Figure 4). For HPM, only S-DCT was observed, with V_max_ and K_m_ values of 0.15 ± 0.01 nmol/mg protein/min and 11.08 ± 2.15 µM, respectively, indicating a significantly lower metabolic capacity in HPM compared to HLM (Figure 4). To compare the intrinsic clearance, the V_max_/K_m_ value was calculated (Figure 4), and it showed that as for S-DCT, RLM and HLM were significantly higher than HPM, with V_max_/K_m_ of 2.23 ± 0.56, 3.90 ± 0.13 to 0.01 ± 0.00 (<0.001) mL/mg protein/min. And to S-DDCT, RLM had a V_max_/K_m_ of 0.28 ± 0.05 mL/mg protein/min and HLM was 0.22 ± 0.02 mL/mg protein/min. It also demonstrated different metabolic ability of different microsomes to different metabolites. The proportion of SCT metabolites was further analyzed (Figure 5). In RLM, approximately 3.36% of SCT was metabolized, with 6.30% of S-DCT further demethylated to form S-DDCT. In HLM, 8.46% of SCT was metabolized, and 4.74% of S-DCT subsequently underwent demethylation. While in HPM, only 0.04% of SCT was metabolized to S-DCT.

Since SCT is an effective and relatively safe SSRIs to treat MMD, it is widely used in clinical treatment. Although there are some methods to detect SCT and its metabolites in human plasma (Hu et al., 2022), no method has been developed to test its placental metabolism. As MDD is a serious disease during pregnancy, it is essential and urgent to develop an effective and precise SCT placental detection method. As a classic model for *in vitro* drug metabolism research, human placental microsomes play a core role in simulating the metabolic environment in placental tissue. Since the placenta is a key barrier for substance exchange between the mother and fetus during pregnancy, studying the metabolism of escitalopram in this model is essential to evaluate whether the drug will affect fetal exposure through placental metabolism. This study is the first *in vitro* demonstration of this metabolic potential in human placental microsomes, although only 0.04% of escitalopram was metabolized to S-DCT in HPM (Figure 5), this indicates that the placenta has metabolic capacity. However, the specific metabolic mechanism of SCT still needs further research.

## 4 Conclusion

This study developed and validated a sensitive LC-MS/MS method for quantitative analysis of SCT, S-DCT, and S-DDCT in RLM, and applied it to HLM and HPM. This method presented high specificity, reliable accuracy and precision, good extraction recovery and stability, and no significant matrix effects. The incubation experiments using these microsomal systems showed that all three types of microsomes can metabolize SCT. Especially, this study has found for the first time that HPM can metabolize SCT. However, compared to RLM and HLM, HPM exhibits significantly lower metabolic capacity and slower metabolic rate.

## Data Availability

The original contributions presented in the study are included in the article/supplementary material, further inquiries can be directed to the corresponding author.
